# Active West Nile virus transmission in Brazil: an epidemiological study

**DOI:** 10.1016/j.lana.2025.101229

**Published:** 2025-09-08

**Authors:** Shirlene T.S. de Lima, Ingra M. Claro, Xinyi Hua, Ronaldo de Jesus, Kyla Serres, Leda M. Simões Mello, Julia Forato, Filipe R.R. Moreira, Rodrigo B. Kato, Gustavo N. Guimarães, Gabriel C. Scachetti, Pamela dos Santos Andrade, Larissa M.F. Duarte, Maria Eduarda T. de Lima, Clarissa P.M. Ferraz, Marisa P.N. Vianna, Rodrigo M. Santiago, Enock L.R. Braga, Igor S. Carneiro, Antonio Carlos L Firmino, Milena G. Cabral, Caio Souza, Liana Perdigão Mello, Sabrina Li, Ester C. Sabino, Maria Anice M. Sallum, Scott C. Weaver, Nuno R. Faria, Camila M. Romano, Simon Dellicour, José Luiz Proenca-Modena, William M. de Souza

**Affiliations:** a Department of Microbiology, Immunology, and Molecular Genetics, College of Medicine, University of Kentucky, Lexington, USA; b Laboratório Central de Saúde Pública do Ceará, Fortaleza, Brazil; c Department of Genetics, Microbiology and Immunology, Laboratory of Emerging Viruses, Institute of Biology, University of Campinas, Campinas, Brazil; d Instituto de Ciências Biológicas, Universidade Federal de Minas Gerais, Belo Horizonte, Brazil; e Spatial Epidemiology Lab, Université Libre de Bruxelles, Brussels, Belgium; f Interuniversity Institute of Bioinformatics in Brussels, Université Libre de Bruxelles, Vrije Universiteit Brussel, Brussels, Belgium; g Departamento de Genética, Universidade Federal do Rio de Janeiro, Rio de Janeiro, Brazil; h Laboratório Central de Tecnologias de Alto Desempenho em Ciências da Vida, University of Campinas, Campinas, Brazil; i Instituto de Medicina Tropical, Faculdade de Medicina da Universidade de São Paulo, São Paulo, Brazil; j Departamento de Patologia, Faculdade de Medicina da Universidade de São Paulo, São Paulo, Brazil; k Departamento de Epidemiologia, Faculdade de Saúde Pública, Universidade de São Paulo, Brazil; l College of Medicine, Universidade de Fortaleza, Fortaleza, Brazil; m School of Geography, University of Nottingham, Nottingham, UK; n Department of Microbiology and Immunology, University of Texas Medical Branch, Galveston, USA; o World Reference Center for Emerging Viruses and Arboviruses, University of Texas Medical Branch, Galveston, USA; p Department of Infectious Disease Epidemiology, MRC Centre for Global Infectious Disease Analysis, School of Public Health, Imperial College London, London, UK; q Hospital das Clínicas da Faculdade de Medicina da Universidade de São Paulo, São Paulo, Brazil; r Department of Microbiology, Immunology and Transplantation, Laboratory for Clinical and Epidemiological Virology, Rega Institute, KU Leuven, Leuven, Belgium

**Keywords:** West Nile virus, Arbovirus, Vector-borne viruses, Mosquito-borne flavivirus, Emerging viral diseases, Flavivirus

## Abstract

**Background:**

West Nile virus (WNV) is a mosquito-borne flavivirus that can cause neurological and fatal disease in animals and humans. Since its introduction into the USA in 1999, WNV has become the leading arbovirus in North America. In contrast, no major WNV outbreak has been reported in South America. Our study investigated active WNV circulation in Brazil.

**Methods:**

We examined WNV epidemiological, molecular, genomic, and serological data from Brazil from January 2014 to December 2024. We also conducted WNV testing in 561 patients with febrile illness, neuroinvasive disease, or death between January 2019 and January 2024 in Ceará State, Brazil. Next, we conducted time series, mapping, ecological niche modeling, age-sex distribution, phylogenetic analyses, and statistical hypothesis tests.

**Findings:**

Between January 2014 and December 2024, 110 West Nile cases were reported from 13 of 27 Brazilian states. In addition, our retrospective study in Ceará State revealed 12.1% (68 of 561 patients) were WNV cases, peaking in 2023, when 42.6% (29 of 68) of cases occurred. Among WNV cases, 7 (10.3%) had detected WNV RNA in serum, cerebrospinal fluid, or both, whereas 62 (89.7%) were IgM-positive, with 29 presenting with neurological complications, 35 with febrile illness, and four fatalities. WNV cases were reported in all months, with the highest numbers between May and August. Most cases were female (female-to-male ratio, 1.1:1), and the median age of patients was 40 years (interquartile range, 20–57). Our phylogenetic analysis showed that WNV lineage 1a circulated in Ceará State and caused a fatal horse case. Our ecological niche models identified several areas, mainly situated in the Northeast region, linked to a potentially higher risk of human exposure to local WNV circulation.

**Interpretation:**

These findings comprehensively described consistent WNV circulation in Brazil and may contribute to informing public health policy, focusing on the strategies to determine the WNV burden in South America.

**Funding:**

10.13039/100000861Burroughs Wellcome Fund, 10.13039/100010269Wellcome Trust, 10.13039/100000002National Institutes of Health, 10.13039/501100001807São Paulo Research Foundation, Brazilian Ministry of Science, and Brazilian National Council for Scientific and Technological Development.

**Research in context**

**Evidence before this study**

West Nile virus (WNV) is a re-emerging mosquito-borne flavivirus that is a significant public health concern nearly worldwide. Since its introduction into the United States of America in 1999, WNV has become the leading mosquito-borne human pathogen in North America. In contrast, few WNV cases have been reported in South America, despite suitable ecological conditions for transmission. We searched PubMed without language restrictions from the database up to May 16, 2025, for studies published with the terms “West Nile virus South America” or “West Nile virus Brazil”. We found several cross-sectional serological studies that indicated previous WNV infections in humans, birds, and equids in South America. However, no major outbreaks have been reported, and WNV cases were mostly restricted to equines. To our knowledge, there have been no reports that investigate active WNV infection in humans using a longitudinal approach in South America.

**Added value of this study**

We combined Brazil's national epidemiological data and a six-year longitudinal molecular and serological study in Ceará State, Brazil, to identify the continued circulation of WNV in humans across 11 of 27 Brazilian states between 2014 and 2024. These cases included individuals with febrile illness, neurological presentations, and fatal outcomes. Our study also confirmed the presence of WNV lineage 1a in Ceará State since June 2019, and we estimated the introduction of WNV into Brazil to be around 2007. Additionally, we produced a risk map of human exposure to WNV in Brazil, contributing to national-level surveillance efforts.

**Implications of all the available evidence**

Our findings indicated established WNV transmission to humans and equids in several Brazilian states. These results highlight the need for Brazilian public health authorities to strengthen WNV diagnosis as a potential cause of febrile illness, neurological disease, and unexplained deaths, particularly in areas co-endemic with other arboviruses such as dengue, chikungunya, and Zika. Our findings emphasize the necessity for increased testing to more accurately map and mitigate the risk of human exposure to WNV.

## Introduction

West Nile virus (WNV) is a re-emerging mosquito-borne flavivirus that is a significant threat to human and animal health nearly worldwide.^1^ WNV transmission occurs in a complex enzootic cycle involving hematophagous mosquitoes (mainly *Culex* species) and birds, with humans and equids being incidental and dead-end hosts.^1^ WNV is endemic in several countries in Africa, North America, and Europe, and geographic expansion has been associated with changes in ecological suitability, due to climate and anthropogenetic changes.^2^^,^^3^ WNV infection in humans is commonly asymptomatic, but 20% of infected people develop a febrile illness, and less than 1% develop central nervous system (CNS) disease, which can lead to permanent sequelae, including physical, neurologic, and cognitive disabilities.^1^^,^^4^ At present, no vaccines or antiviral drugs are available to prevent WNV infection or treat West Nile disease in humans, but there are two vaccines available to prevent disease in horses in the United States of America (USA) and Canada.

Soon after its introduction into New York City, USA, in 1999, WNV spread across the USA.^5^^,^^6^ Currently, WNV is the major mosquito-borne disease in the USA, where over 7 million people are estimated to have been infected since its introduction.^4^^,^^7^ WNV can also cause ecological disruption because it can be fatal for many native wild bird species.^8^ Between 2001 and 2004, WNV spread to Central America and the Caribbean, probably carried by migrating birds.^9^ In South America, the first WNV cases reported were three horses that died in Argentina in April 2006.^10^ Later, WNV was isolated in 2012 from samples from captive flamingos in Colombia.^11^ Since then, there have been few reported cases in Latin America, despite serological evidence of ongoing transmission.^12^^,^^13^ Most Latin American countries have suitable conditions for enzootic and epidemic WNV transmission, including susceptible human and equine populations, transmission-competent *Culex* mosquitoes, warm temperatures, and migratory bird routes connected with North America, where the WNV remains enzootic/endemic.

Between 2014 and 2022, 13 serologically confirmed WNV human cases were reported in Piauí and Tocantins States, Brazil. WNV was also isolated or detected in horses with neurological disease in Bahia, Ceará, Espírito Santo, São Paulo, Minas Gerais, and Piauí States from 2018 to 2020.^14^, ^15^, ^16^ Furthermore, WNV was isolated from *Culex* spp. mosquitoes in Pará State in March 2017.^17^ Additionally, numerous serosurveys indicated WNV transmission across Brazil’s five geographic regions.^12^, ^13^, ^14^, ^15^ However, the active transmission dynamics of WNV in Brazil remain understudied. Here, we contextualize and analyze the spread of WNV in Brazil from January 2014 to December 2024 and combine epidemiological, molecular, serological, and genomic analyses to investigate circulation between January 2019 and January 2024 in Ceará State, Brazil.

## Methods

### Study design and participants

Our epidemiological study combined WNV genome sequencing, molecular, serological, and clinical data from across Brazil. National epidemiological data on West Nile cases were obtained from the Brazilian Ministry of Health (Supplementary material p 1). This dataset included the individualized data of West Nile cases from January 1, 2014, to December 31, 2024. We also conducted a retrospective, longitudinal molecular and serological study between January 2019 and January 2024 in Ceará State, Northeastern Brazil. Sera and cerebrospinal fluid (CSF) samples were taken from patients presenting at primary health care units with acute febrile illness and/or neurologic disease suspected to be caused by arbovirus infection. Additionally, we included sera, CSF and tissue samples (i.e., brain, heart, lung, liver, spleen, and kidney) from fatal cases with clinically suspected arboviral infection recorded by the Death Verification Service of the State Health Secretariat of Ceará State. These data included de-identified information, such as age, sex, municipality of residence, date of symptom onset, and date of sample collection. All procedures followed the ethical standards of the responsible committee on human experimentation and were approved by the ethics committees from the University of Campinas, Brazil (no. #53910221.0.0000.5404). Informed consent was waived for the use of residual samples originally collected for diagnostic purposes. In addition, we included novel WNV genomic data obtained from a brain sample from a fatal horse case from Boa Viagem municipality in Ceará State on June 4, 2019, as previously reported.^18^

### Procedures

Residual diagnostic sera, CSF, and tissue samples were collected from patients with acute febrile illness, neuroinvasive disease, or death, all with primary suspected arboviral infection. Basic clinical and demographic data were collected through the Brazilian Laboratory Environment Management System. Tissue fragments of 1 cm^3^ from autopsies were homogenized using a mortar and pestle with 15 ml of Leibovitz's L-15 medium (Gibco, USA) supplemented with 10% Fetal Bovine Serum (FBS) (Gibco, USA) and 1% antibiotic-antimycotic (Gibco, USA). Then, the samples were centrifuged at 1500×*g* for 15 min at 4 °C, and the supernatant and pellet were separated. Viral RNA was extracted from homogenized tissue, serum, and CSF samples using the Extracta DNA and RNA Kit (Loccus, Brazil). WNV RNA was detected using RT-qPCR (Supplementary appendix p 1). RNA samples positive for WNV by RT-qPCR were submitted for WNV genome sequencing by Illumina and Nanopore sequencing approaches (Supplementary appendix p 1–2). Next, we appended the new WNV genome with 70 other WNV complete coding sequences available in GenBank from database inception to May 20, 2025, and we conducted phylogenetic analysis (Supplementary appendix p 2). Additionally, a subset of serum and plasma samples was submitted to detect human antibodies specific for WNV using the WNV anti-IgM ELISA Kit (Creative Diagnostics, USA). All the samples were tested in duplicate. Samples WNV positive by RT-qPCR or IgM ELISA were tested for CHIKV, ZIKV, and DENV by RT-qPCR and IgM ELISA for DENV, CHIKV, and ZIKV (Supplementary appendix p 1).

### Statistical analysis

All analyses were carried out in RStudio version 4.4.0 (https://posit.co). Incidences were calculated based on the 2022 Brazilian population census reported by the Institute of Geography and Statistics (www.ibge.gov.br). The correlation coefficients of optical density (OD) values and the interval between symptom onset and sample collection were determined by Pearson's correlation coefficient. The differences in mean OD values between outcome groups (febrile illness versus neuroinvasive) and between sexes (male versus female) was calculated using an unpaired t-test. The differences in OD values among age groups were assessed using the Kruskal–Wallis rank sum test, followed by Dunn’s test for multiple comparisons. We also employed a boosted regression trees (BRT) approach to model the risk of local WNV circulation leading to human infections across Brazil. We trained 100 BRT models at the municipality level using confirmed human cases as presence data, exploiting negative test data to inform the preferential sampling of pseudo-absences, and considering 14 environmental factors tested for their association with the risk of local WNV circulation. The predictive performance of each replicate analysis was evaluated using the area under the receiver operating characteristic curve (AUC), where high predictive performance was indicated by values near one and values around or below 0.5 reflect low predictive performance (Supplementary appendix p 2–3).

### Role of the funding source

The funders of the study had no role in study design, collection, analysis interpretation of data, and writing of the report.

## Results

**Fig. 1:**
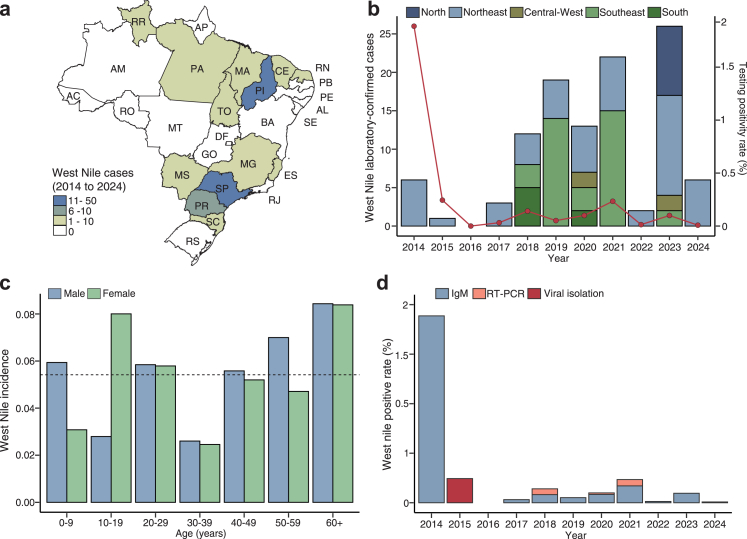
**West Nile cases in Brazil. (a)** Map colored according to the number of probable and confirmed West Nile cases per state in Brazil between January 1, 2014, and December 31, 2024. **(b)** Number of acute West Nile cases per year in all five Brazilian regions between January 1, 2014, and December 31, 2024. The red line indicates the positive testing rate. The detailed testing positive rate is shown in Supplementary Table S1. **(c)** Cumulative incidence (cases per 100,000 inhabitants) of West Nile based on age–sex distribution. The horizontal dash line indicates an overall incidence of 0.054 per 100,000 inhabitants in Brazil. **(d)** West Nile virus positivity rate by the detection method between January 1, 2014, and December, in Brazil. AC = Acre. AL = Alagoas. AM = Amazonas. AP = Amapá. BA = Bahia. CE = Ceará. ES = Espírito Santo. DF = Distrito Federal (Federal District). GO = Goiás. MA = Maranhão. MG = Minas Gerais. MS = Mato Grosso do Sul. MT = Mato Grosso. PA = Pará. PB = Paraíba. PE = Pernambuco. PI = Piauí. PR = Paraná. RJ = Rio de Janeiro. RN = Rio Grande do Norte. RO = Rondônia. RR = Roraima. RS = Rio Grande do Sul. SC = Santa Catarina. SE = Sergipe. SP = São Paulo. TO = Tocantins. IgM = Immunoglobulin M. RT-PCR, reverse transcription polymerase chain reaction.

Between January 1, 2014, and December 31, 2024, 110 West Nile cases (i.e., probable and confirmed) were reported to the Brazilian Ministry of Health from 59 municipalities across 13 of 27 Brazilian states (Fig. 1a). During this period, 194,445 suspected West Nile cases were tested for WNV, with a positivity rate of 0.06%. The median annual number was 6 cases (interquartile 2.5–16), with an increase in the number of cases up to 2023, followed by a substantial decline in 2024 (Fig. 1b). Piauí State had the highest number of cases (40.9%, 45 of 110), with a cumulative incidence of 1.4 cases per 100,000 inhabitants from 2014 to 2024 (Fig. 1a). The cases occurred throughout the year, but 48.2% (53 of 110) occurred between May and August. We found a female-to-male ratio of 1.1:1, and an overall incidence of 0.054 cases per 100,000 inhabitants (Fig. 1c). Higher incidences were observed in males between the ages of 50 and 59 and females between 10 and 19, and in both sexes over 60 years old (Fig. 1c). No significant sex and age group differences were observed with two-way ANOVA test (p = 0.9474 and 0.2429, respectively). WNV cases were predominantly identified by detection of IgM against WNV (86.4%, 95 of 110) (Fig. 1d).

Next, we focus our investigation on Ceará State due to a fatal horse case of WNV in Ceará State on June 4, 2019.^18^ For this, we used RT-qPCR and ELISA to investigate acute WNV infection in 561 patients who sought care in the public health care service between January 2019 and January 2024 in Ceará State, Brazil. A total of 555 patients were residents from 86 out of 184 (46.7%) municipalities in Ceará State, Brazil. Additionally, we included samples from 6 patients who attended primary health care units in Ceará State but resided in other Brazilian states. Among the patients included were 410 neuroinvasive cases, comprising 60 patients with only serum samples, 90 patients with only CSF samples, and 260 with paired CSF and serum samples. Also, we screened CSF, serum, and multiple tissues (i.e., brain, heart, lung, liver, spleen, and kidney) from 90 patients who died of suspected arboviral infection. Additionally, we included serum samples from 55 patients with acute febrile illness between January 2022 and January 2024.

**Fig. 2:**
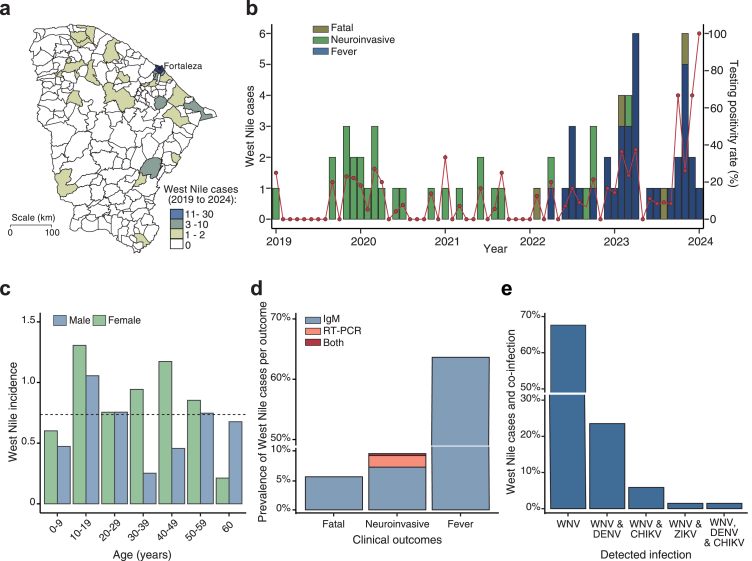
**West Nile cases in Ceará State, Brazil. (a)** Spatial distribution of probable and confirmed, acute West Nile cases per municipality (n = 184 municipalities) in Ceará from January 2019 to January 2024. **(b)** Number of probable and confirmed, acute West Nile cases per month from January 2019 to January 2024. The red line indicates the positive testing rate. **(c)** Cumulative West Nile incidence (cases per 100,000 inhabitants) based on age–sex distribution. The horizontal dash line indicates an overall incidence of 0.736 per 100,000 inhabitants in Ceará State. **(d)** The proportion of acute WNV cases per clinical outcomes. **(e)** Monoinfection and co-infection of WNV and other endemic arboviruses in Ceará State, Brazil.

We identified 12.1% (68 of 561) of these cases as probable or confirmed West Nile between March 2019 and January 2024, peaking in 2023 with 42.6% (29 of 68) of cases (Fig. 2a). We found that West Nile cases occurred throughout the year, with higher frequencies in November and March (Fig. 2b). A total of 67 WNV cases was identified from 27 municipalities across Ceará State, Brazil. Of these, 43.3% (29 of 67) of these cases lived in Fortaleza City, the state's most populous municipality with 2.7 million inhabitants (Fig. 2a). Also, acute WNV infection was detected in a patient residing in São José de Ribamar municipality, located in Maranhão State, Northeast Brazil. The WNV cases were predominantly female (female-to-male ratio, 1.3:1) and the median age of patients was 26 years (interquartile range, 17–43) (Fig. 2c). No significant sex and age group differences were observed with two-way ANOVA test (p = 0.2456, 0.3563, respectively).

All West Nile cases were identified by RT-qPCR and/or detection of WNV-specific IgM antibodies (Fig. 2d). We detected RNA WNV in 1.2% (7 of 561) neuroinvasive cases between January 2021 and October 2022. WNV RNA was identified in both CSF and serum samples from four patients, while three cases were positive only in CSF. The cycle threshold (Ct) values obtained by real-time RT-qPCR-positive ranged between 33.4 to 38.8, indicating low viral loads. All West Nile cases by RT-qPCR were negative for CHIKV, ZIKV, and DENV by RT-qPCR and serological assays. No WNV RNA was detected in samples from patients with acute febrile illness or from deceased individuals.

By measuring IgM antibodies specific against WNV, we found a seropositivity rate of 14.1% (62 of 439) between January 2021 and January 2024. The highest IgM-positive rate (63.6%, 35 of 55) was observed in patients with acute febrile illness, and neuroinvasive cases or deceased patients with suspected arboviral infection presented IgM-seropositivity of 7.4% (23 of 311) and 5.5% (4 of 73), respectively (Fig. 2d). Of 62 cases positive for WNV IgM, 25.8% (16 of 62) were co-positive for DENV IgM antibodies, and 8.1% (5 of 62) were IgM-positive for CHIKV (Fig. 2e). We also detected DENV RNA in a patient IgM-positive for WNV. Also, one of the four patients with WNV RNA in CSF and serum also presented WNV-specific IgM antibodies. We found a very weak and non-significant correlation between ELISA OD values and the interval between symptom onset and sample collection (Spearman’s ρ = 0.0981, p = 0.4721). Additionally, no statistically significant difference was observed between the OD values between neurological and febrile illness cases (unpaired t-test, p = 0.8333), sexes (unpaired t-test, p = 0.4051), or age groups considering the ranges below 18 years, between 18 and 60 years, and over 60 years old (Kruskal–Wallis rank sum test, p = 0.1173).

**Fig. 3:**
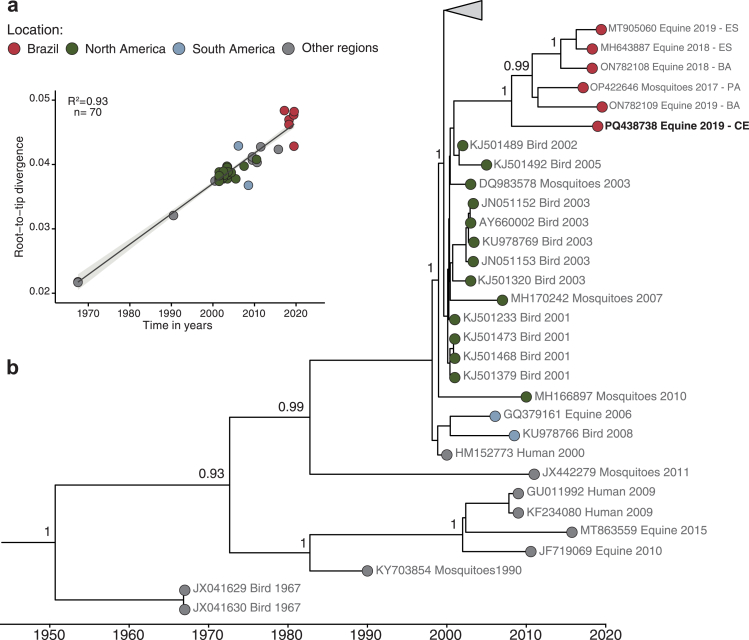
**Phylogenetic analysis of the West Nile virus (WNV) strain in Brazil.** Maximum clade credibility tree of 70 WNV genomes from the WNV lineage 1a, including the new WNV genome from a fatal horse case in 2019. **(a)** Regression of sequence sampling dates against root-to-tip genetic distances in a maximum likelihood phylogeny of the WNV lineage 1. **(b)** Sequences are colored according to source locations. An uncorrelated lognormal relaxed molecular clock (UCLD) model with a lognormal distribution on evolutionary rate was used to generate the time-rooted tree. Posterior probability scores are shown next to key well-supported nodes. BA = Bahia. CE = Ceará. ES = Espírito Santo. PA = Pará.

Next, we investigated WNV genetic diversity by attempting genomic sequencing of all RNA from RT-qPCR-positive human cases and one brain tissue sample from an RT-qPCR-positive horse (Ct-value = 19). This fatal horse case from the Ceará State was previously described,^18^ but the WNV genome was not sequenced. We obtained a complete genome sequence only from the equine sample with 91% coverage and a mean depth of at least 1000 × . Sequencing and viral isolation attempts in Vero CCL-81 cells and suckling mice with human samples WNV-positive by RT-qPCR were unsuccessful (Supplementary appendix p 2). Our phylogenetic analysis revealed that this WNV sequence from Ceará State belonged to WNV lineage 1a and clustered with other Brazilian WNV genomes in a single clade with strong statistical support (bootstrap value = 100%) (Fig. 3a and b). Root-to-tip regression of genetic divergence against sampling times confirmed a well-supported temporal signal in our genomic dataset (R^2^ = 0.93, Fig. 3a). We then conducted molecular clock analyses using 70 WNV genomes, including the six WNV strains from Brazil, to estimate the evolutionary history of WNV in the Americas. WNV strains identified in Brazil shared a common ancestor with strains circulating in North America (i.e., United States of America and Mexico) and formed a well-supported cluster with posterior probability support (Fig. 3b), indicating a single introduction of lineage 1a into Brazil from North America. The Brazilian WNV strains shared a common ancestor around late 2007 (95% Bayesian credible interval 2002 to 2012) (Fig. 3b), indicating that WNV may have been circulating cryptically for at least 5 years in Brazil. The available strains from Argentina (GenBank number GQ379161) and Colombia (GenBank number KU978766) formed a separate cluster that was not phylogenetically linked to the Brazilian cluster, suggesting another introduction in South America from North America. No evidence of recombination was found in WNV strains identified in Brazil.

We then used a BRT approach to estimate the WNV ecological suitability across Brazil. Our ecological niche models were associated with a relatively good predictive performance with an average AUC of 0.82; 95% credible interval = 0.77–0.85. Our ecological estimates indicate that the Northeast region—and the Ceará state in particular—had the highest estimated ecological suitability for WNV, followed by the Southeast Region, mainly the São Paulo and Minas Gerais states (Supplementary Fig. S1).

## Discussion

In our epidemiological study, we have provided a longitudinal assessment of WNV circulation in humans in Brazil between 2014 and 2024, and investigated active WNV circulation in Ceará State. We described the detection of 110 human West Nile cases (i.e., probable and confirmed) from 2014 to 2024 in 13 of 27 Brazilian states. We also identified 68 West Nile cases (i.e., probable and confirmed) among patients with neurological presentations, febrile illness, and fatal outcomes over a six-year period in multiple municipalities in Ceará State. We identified WNV lineage 1a in the Ceará State and caused a fatal horse case. The continued and widespread distribution of West Nile cases indicated sustained transmission in Brazil at a lower level, which contrasts with the absence of major West Nile outbreaks elsewhere in South America.^19^ Several factors could explain this discrepancy, such as human WNV infections are often oligosymptomatic or asymptomatic (up to 80%), leading to an underestimation of the WNV presence.^1^ Also, Brazil is endemic for multiple arboviruses (i.e., DENV, CHIKV, ZIKV), with similar febrile clinical presentations as symptomatic WNV infections,^20^ making cases of acute febrile illnesses more likely to be misdiagnosed by clinical-epidemiological criteria. Furthermore, WNV-infected patients experience brief and lower viremia (1–3 days), with infectious virus rarely isolated from serum or CSF,^21^ which also explains our limitation in isolating and sequencing the WNV from human cases. Therefore, strengthening WNV diagnostic capacity within the national surveillance program, particularly for neuroinvasive cases, could significantly improve the understanding of the West Nile burden in Brazil.

Our findings regarding sex distribution show that West Nile affected more females than males in Ceará State, which differs from data observed at the national level in Brazil (54%, 61 of 113), as well as in the USA, where males accounted for 59% of cases reported between 2009 and 2018.^7^ Importantly, we found that the median age of West Nile patients in Brazil was lower than in the USA. In Ceará State, most cases occurred in individuals around 30 years old, and at the national level in Brazil, the median was 41 years old, whereas in the USA, the majority of cases are reported in individuals over 60 years old.^7^ WNV infection can cause severe disease, particularly in elderly individuals or those with underlying health conditions. In Brazil, the aging population, coupled with the high prevalence of some comorbidities that are risk factors for West Nile neuroinvasive disease,^22^ may contribute to the growing concern for severe West Nile disease in the coming years. For example, the number of Brazilians aged 65 years and older increased by 57% from 14.1 million in 2010 to 22.2 million in 2022, now representing 11% of the total population, and hypertension and diabetes mellitus affect nearly 60 and 12 million people, respectively.^23^, ^24^, ^25^

We found that the West Nile cases occurred mainly between November and March in Ceará State, coinciding with the rainy season and increased temperatures in the Northeast region, where Piauí and Ceará States are located. Similarly to other subtropical areas such as Namibia and South Africa, the risk of WNV transmission is highest from October to May,^26^ when both temperature and precipitation are higher. These ecological factors are important for mosquito-borne diseases because they can contribute to the magnitude and seasonality of transmission by affecting mosquito reproduction, survival, biting rates, short extrinsic incubation period, and adult vector population density.^27^ Additionally, this period overlaps with the arrival of migratory birds from the Northern Hemisphere on the Coast of Northeast Brazil, and these birds could have potentially contributed to previous or new introductions of WNV in the region. Further molecular surveillance studies of migratory birds along the five migratory routes in South America are crucial, particularly focusing on coastal Northeast Brazil to elucidate their role in WNV introductions.

The outcome of our ecological niche modeling in Brazil should be interpreted with caution. First, relative influences and partial dependency plots do not really align with trends observed in previous studies based on a similar approach.^28^^,^^29^ Second, the outcome of such an ecological niche modeling approach will, by essence, remain sensitive to the currently available positive and negative test data. While municipalities without any reported human infection were preferentially sampled as pseudo-absence locations if associated with a relatively higher number of reported negative tests (Supplementary appendix p 3), the testing effort remains highly heterogeneous across the country. For instance, Brazilian states like Paraná and Espírito Santo are associated with a higher level of diagnostic efforts (Supplementary Fig. S1). As a result, local WNV circulation leading to human infections could potentially have been undetected in a series of municipalities associated with a more limited diagnostic effort, which could in turn impact the accuracy of the risk mapping obtained through this ecological niche modeling approach. In this context, more comprehensive WNV case data (i.e., presence and absence data) will be important to future risk mapping analyses. Enhancing surveillance efforts would be particularly important for Ceará, Maranhão, Piauí, Rio Grande do Norte, Tocantins, Minas Gerais, Espírito Santo, São Paulo, Paraná, where reported human and/or horse WNV cases indicated continued viral circulation.^14^^,^^15^ Furthermore, current vector control efforts targeting anthropophilic *Aedes aegypti*, the primary vector for CHIKV, DENV, and ZIKV, should be expanded to include ornithophilic *Culex* species, particularly *Cx. quinquefasciatus*, which is also abundant in urban habitats. *Cx. quinquefasciatus* is widely distributed in Brazil, has been demonstrated to be a competent vector for the WNV strain circulating in the country,^30^ including by detection of RNA WNV followed by viral isolation in 2017.^17^ Additionally, WNV should be considered as a differential diagnosis for CNS infections in equines, especially fatal cases negative for rabies, western-, eastern-, and Venezuelan-equine encephalitis viruses.

There are several limitations to our study. First, we were not able to obtain WNV genomic sequences from human WNV cases, which prevented us from confirming that the WNV lineage 1a identified in horses is also responsible for human infections. Here, we propose continued molecular surveillance, including mosquitoes, to obtain samples with a higher viral load from which WNV genome sequences can likely be obtained. Second, the presence of multiple flaviviruses (e.g., DENV and ZIKV) in Ceará State may generate cross-reactivity in serological assays. Although some WNV IgM–positive samples could result from cross-reactions with other circulating flavivirus in the region, the frequency of IgM cross-reactivity among flaviviruses is significantly lower than that for IgG antibodies.^31^ Third, we have no follow-up information on neuroinvasive and febrile WNV cases and information on individual-level factors related to socio-economic status can be important determinants of arboviral risk. Fourth, our study was limited to passive syndromic surveillance of symptomatic healthcare-seeking cases, and we were not able to capture the oligo- and asymptomatic cases. Further systematic blood donor screening could be a valuable approach to address this limitation.^32^ Finally, continued entomological and ornithological surveillance is needed to better elucidate the WNV transmission dynamics in Brazil.

In conclusion, our study identified the continued WNV circulation by identifying human patients with neurological diseases, fatal cases, and febrile illness in Brazil. Our findings provided an important context about WNV transmission in South America and may inform public health policy focusing on the strategies to determine the West Nile burden through continuous laboratory diagnosis of WNV to determine its prevalence in humans and equines in Brazil, as well as public health countermeasures such as mosquito control in those areas.

## Contributors

WMdS and STSdL conceptualized the study. STSdL, IMC, LMSM, JF, GNG, GCS, PSA, LMFD, METdL, CPMF, MPNV, RBK, RMS, ELRB, ISC, MGC, CS, LPM contributed to the acquisition of data. STSdL, IMC, XH, RBK, RdJ, FRRM, ACLF, SL, KS, SD, and WMdS contributed to the data analysis. WMdS, STSdL, IMC, XH, FRRM, KS, SCW, SD, NRF, and JLPM contributed to data interpretation. WMdS drafted the manuscript. WMdS, STSdL, ECS, SL, MAMS, NRF, CMR, SCW, KS, SD, and JLPM revised the manuscript. Funding: WMdS, ECS, NRF, CMR, SD, and JLPM acquired funding for the study. All authors read and approved the final version of the manuscript and had access to all the data in the study. WMdS and STSL accessed and verified all the data reported in the study.

## Data sharing statement

All statistical computing analyses were conducted using the R project. R packages necessary for analysis and visualization include: tidyverse, raster, tmap, scico and sf. No custom code was developed. The new sequence was deposited in GenBank with accession number PQ438738.

## Editor note

The Lancet Group takes a neutral position with respect to territorial claims in published maps and institutional affiliations.

## AI statement

The authors declare no use of generative AI or AI-assisted technologies in this study.

## Declaration of interests

The authors declare no competing interests.

## Appendix A Supplementary data

Supplementary Appendix, Fig. S1,andTables S1–S4

Abstract_Portuguese_final
