# Supplementary material for: Active West Nile virus transmission in Brazil: an epidemiological study
Source: Lancet Reg Health Am. 2025 Sep 8;51:101229. doi: 10.1016/j.lana.2025.101229 (PMC12448031; doi:10.1016/j.lana.2025.101229)
Supplement: Supplementary Appendix, Fig. S1,andTables S1–S4 [file mmc1.docx]

**Supplementary appendix**

**Materials and Methods**

**Data source and definition of cases**

National epidemiological data on laboratory-confirmed West Nile cases were obtained from the Brazilian Ministry of Health, which provides continuous laboratory diagnosis through the Brazilian universal healthcare system (SUS, *Sistema Único de Saúde*) across all 27 federal units. West Nile cases in humans and animals (equines and birds) has been nationally notifiable disease since September 28, 2017. The suspected neuroinvasive cases by arboviruses are defined by the Brazilian Ministry of Health as acute cases of encephalitis, myelitis, encephalomyelitis, polyradiculoneuritis (Guillain-Barré syndrome), or other central or peripheral neurological syndromes diagnosed by neurologists, neuropediatrician or infectologist, in the absence of a more likely clinical explanation^1^. We defined confirmed West Nile cases as positive by reverse-transcription quantitative polymerase chain reaction (RT-qPCR) for WNV, and probable West Nile cases by immunoglobulin M (IgM) detection for WNV. Furthermore, laboratory-confirmed cases of chikungunya, dengue, or Zika were defined as a patient with one positive laboratory result for chikungunya (CHIKV), dengue (DENV), or Zika (ZIKV) viruses, either by RT-qPCR, IgM detection, viral isolation, and/or non-structural protein 1 (NS1) antigen for DENV. No clinical epidemiological cases were included in this study.

**Real-time Quantitative Reverse Transcription-Polymerase Chain Reaction for West Nile, *Chikungunya, Dengue, and Zika Viruses***

Viral RNA was extracted from homogenized tissue, serum, and CSF samples using the Extracta DNA and RNA Kit (Loccus, Brazil) following the manufacturer's instructions. The extracted RNA was then tested by real-time RT-qPCR targeting WNV^2^, CHIKV^3^, DENV serotypes 1 to 4^4,5^, and ZIKV^6^ using the TaqMan™ Fast Virus 1-Step Master Mix (Cat no. 4444434, Applied Biosystems, USA) on the StepOnePlus™ Real-Time PCR System (Applied Biosystems, USA). The RT-qPCR positive samples were defined as threshold cycle ≤39. The primers and probes used for the viral detection are described (Table S2).

**IgM ELISA assay for West Nile, chikungunya, dengue, and Zika virus**

Serum samples were tested using IgM-capture enzyme-linked immunosorbent assays (ELISAs) for antibody response against WNV (Cat no. DEIA1982M, Creative Diagnostics, USA), CHIKV (EI 293a-9601, Euroimmun, Germany), DENV (Cat no. 01PE20/01PE21, Panbio, Abbott, USA), and ZIKV (EI 2668-9601 M, Euroimmun, Germany). An IgM-specific WNV sample was defined as positive if the ratio was ≥1.1.

**West Nile virus genome sequencing and assembly genome**

Positive RNA samples by RT-qPCR (i.e., human and equine samples) were submitted for WNV genome sequencing using a targeted multiplex PCR scheme and the MinION platform (Oxford Nanopore Technologies, UK), as described elsewhere^7^. PCR products were cleaned using AmpureXP purification beads (Cat no. A63882, Beckman Coulter, UK) and quantified using fluorimetry with the Qubit dsDNA High Sensitivity assay on the Qubit 3·0 instrument (Cat no. Q32851, Life Technologies, USA). Amplicons from each sample were normalized, pooled, and barcoded using the Native Barcoding Kit 96 kit (Cat no. EXP-NBD 196, Oxford Nanopore Technologies, UK). Next, sequencing libraries were generated using the SQK-LSK109 Kit (Oxford Nanopore Technologies, Oxford, UK) and were loaded onto an R9·4·1 flow-cell (Oxford Nanopore Technologies, UK). Then, FAST5 files containing the raw signal data were base-called, demultiplexed, and trimmed using Guppy version 4·4·1 (Oxford Nanopore Technologies, UK). The reads were aligned against the WNV strain BeAn854747 (GenBank accession no. MH643887) using minimap2 version 2·17·r941 ^8^ and converted to a sorted BAM file using SAMtools ^9^. Length filtering, quality testing, and primmer trimming were performed for each barcode using guppyplex. Variants were detected with *medaka_variants* and the consensus sequence were built using margin_medaka_consensus (Oxford Nanopore Technologies, UK). Genome regions with a depth coverage below 100-fold were represented with “N” characters. We also submitted the positive RNA samples by RT-qPCR for the viral metagenomic sequencing using the xGen™ ssDNA & Low-Input DNA Library Preparation Kit (Cat no. 10009859, IDT, USA) with MiSeq Reagent Kit version 3 with 600 cycles paired-end (Cat no. MS-102-3003, Illumina, USA) in MiSeq Illumina equipment, following the manufacturer's instructions.

**Phylogenetic analysis**

The novel WNV genome with >91% coverage was generated and aligned with related representative WNV strains with complete coding sequences available in the GenBank database as of June 20, 2024. Then, we performed a multiple sequence alignment (MSA) built using MAFFT version 7·450^10^, and manual adjustment was conducted using Geneious Prime 2023·0·4. The dataset was screened for recombination events using all available methods in RDP version 5^11^. A maximum likelihood (ML) phylogeny tree was performed using IQ-TREE version 2 under a GTR + I + γ model determined by ModelFinder^12,13^. The ultrafast-bootstrap approach with 1,000 replicates was used to determine the statistical support for nodes in the ML phylogeny^14^. Then, regressed root-to-tip genetic divergence was estimated against sampling dates to examine the temporal signal and identify sequences with low data quality of our datasets, such as assembly errors, sample contamination, data annotation errors, sequencing, and alignment errors^15^. No obvious outliers were identified. The dated phylogenetic tree was estimated using BEAST version 1·10·5 under a GTR + I + γ model^16^, an uncorrelated relaxed molecular clock (UCLN) model with a lognormal distribution on evolutionary rate, and a non-parametric coalescent Skygrid tree prior^17^ with 94 grids, one per year since the root of the tree, as estimated from the x-intercept from the root-to-tip regression. This analysis also deployed the Hamiltonian Monte Carlo operator for the Skygrid model^18^. The BEAGLE library was used to enhance computation speed^19^. Lastly, the evolutionary analyses were performed in 10 separate runs of 100 million generations, sampling parameters and trees every 10,000 steps. Maximum clade credibility summary trees were generated using TreeAnnotator version 1·10·69. The phylogenetic tree was visualized using Figtree 1·4·2 (http://tree.bio.ed.ac.uk/software/figtree/).

**West Nile virus isolation**

We attempted to isolate the WNV by inoculating Vero CCL-81 cells with serum and CSF samples that tested positive for RNA WNV using RT-qPCR (Table S2). Briefly, Vero CCL-81 cells were plated in 24-well plates at a concentration of 2·5×10^5^ cells per mL (1·25x10^5^ cells per well) in Minimum Essential Eagle’s Medium (DMEM) supplemented with 10% fetal bovine serum (FBS), and 1% of penicillin of 10,000 units and 10,000 μg/mL streptomycin solution. Subsequently, the CSF and serum samples were diluted 1:10 in DMEM, treated with 2% penicillin and streptomycin, and added to the monolayer. After a one-hour incubation at 37ºC for adsorption, DMEM supplemented with 2·5% FBS and treated with 1% penicillin and streptomycin was added to the monolayer for maintenance. The cells were kept at 37ºC with 5% CO_2_ and monitored for 7 days. Next, the supernatant was collected and subjected to an RT-qPCR assay^2^ to confirm viral isolation, indicated by a decrease in the Ct-value. Also, we performed an attempt of viral isolation in mice knockout for type I interferon receptors (Ifnar-/-) by infection of 4-day-old suckling mice. Mice were injected intracranially with 0.01 mL of patient tissue, and blind passages were made on days 6 to 7 after inoculation. The brain tissue was collected, homogenized, and then subjected to an RT-qPCR assay^2^ to confirm viral isolation. All attempts at viral isolation were unsuccessful.

**Ecological niche modeling analyses**

We employed a boosted regression trees (BRT) approach to model the risk of local WNV circulation leading to human infections across Brazil, using the BRT algorithm implemented in the R package “dismo”. These models were based on the analysis of both presence and absence data. Specifically, all Brazilian municipalities associated with at least one confirmed case of human WNV infection were treated as “presence” locations. For each replicate BRT analysis, we sampled five times as many “pseudo-absence” locations from municipalities with no positive test result but with at least one negative test result. The probability of sampling one municipality as a pseudo-absence location was proportional to the number of negative tests reported for that municipality. With this procedure, we aim to exploit negative test results in order to minimize the risk of sampling pseudo-absences in areas where human WNV infections may be present but remain undetected. For these analyses, occurrence and pseudo-absence data were analyzed along 14 environmental factors known to potentially impact WNV circulation^20,21^. The selected variables include bare vegetation, croplands, grass cover, leaf index, human population density (log-transformed), permanent water, seasonal precipitation, seasonal relative humidity, seasonal temperature, seasonal water, shrublands, soil moisture, tree cover, and urban cover. Data for these environmental factors were obtained from three distinct sources: (i) the Inter-Sectoral Impact Model Intercomparison Project, which focuses on quantifying climate-related risks under various climate change and socio-economic conditions; (ii) ERA5, the fifth generation of the European Centre for Medium-range Weather Forecasts (ECMWF) reanalysis, offering comprehensive global climate and weather information; and (iii) Copernicus Global Land Service (CLS) providing geographical information on land cover and its changes. The contribution of each environmental variable was assessed through the calculation of their relative influence (RI) and the generation of partial dependence plots. RI values were computed by measuring the frequency with which a variable is selected for node splitting across the decision trees. This value was then adjusted based on the squared improvement in model performance associated with each split and averaged over all trees. Additionally, partial dependence plots, or “response curves”, were created to illustrate the variation in ecological suitability attributable to individual predictor variables, with all other variables held constant. To mitigate spatial autocorrelation and associated model overfitting, we implemented a spatial cross-validation method based on block generation and implemented it in the R package “blockCV”, which divides our data into five spatial folds. The BRT models were then trained with the following parameters: a tree complexity of 5, a learning rate of 0·005, and a step size of 10. To ensure robustness, we ran 100 independent BRT replicates and averaged the results for visualization. The predictive performance of each replicate was evaluated using the area under the receiver operating characteristic curve (AUC), where high predictive performance is indicated by values near 1, and values around or below 0·5 reflect low predictive performance.

**Supplementary Figures**


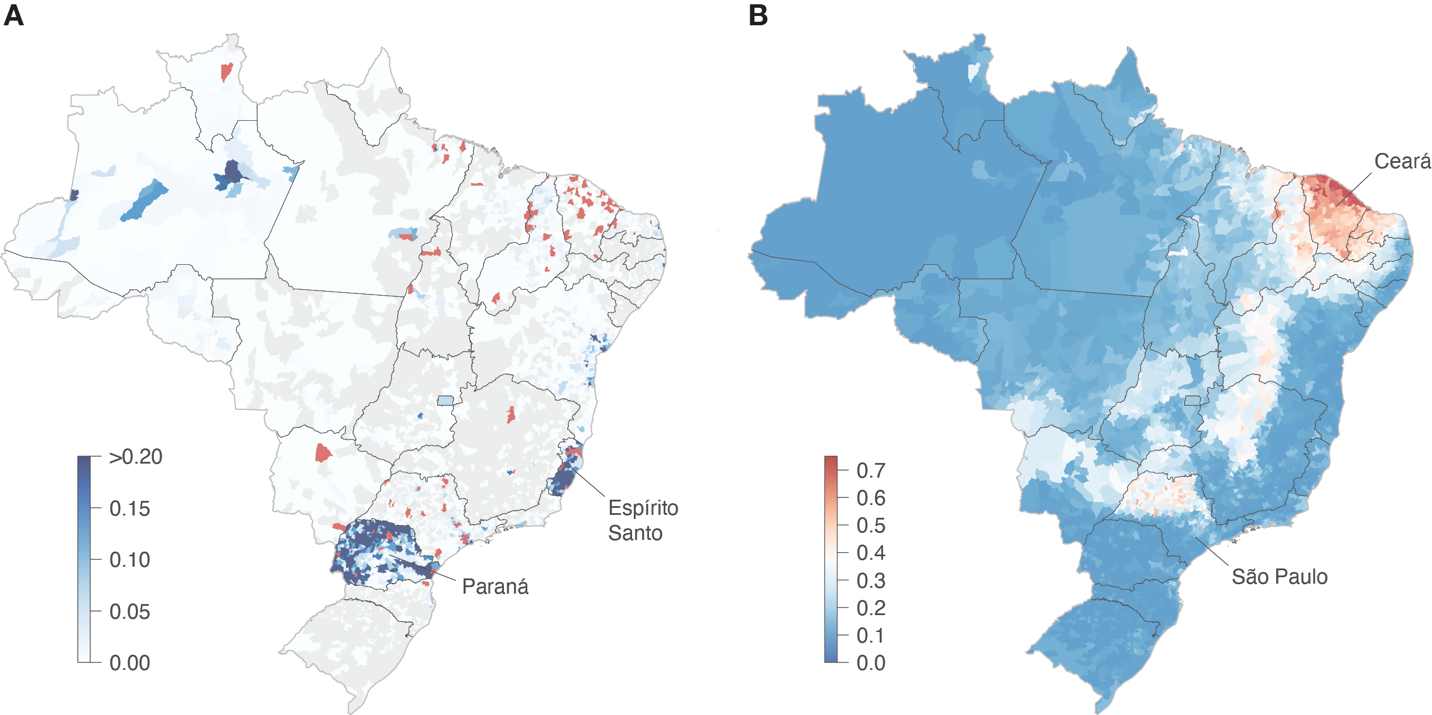


**Fig. S1. Distribution of confirmed human cases of West Nile virus (WNV) infections and estimated ecological suitability for WNV local circulation leading to human infections in Brazil from 2014 to 2023.** (**A**) Displays the distribution of WNV human cases, reporting both positive and negative RT-PCR tests. Municipalities with at least one laboratory-confirmed WNV positive test are colored in red, and municipalities solely associated with laboratory-confirmed negative cases are colored according to a blueish color scale reflecting the number of negative cases per km^2^. (**B**) The estimated ecological suitability for local WNV circulation leading to human infections at the municipality level. This map was obtained by averaging the ecological suitability values predicted by 100 replicate analyses. Dark gray borders indicate the position of the Brazilian state borders. Four Brazilian state names are highlighted because explicitly mentioned in the text when discussing the results of the ecological niche modelling analyses.

**
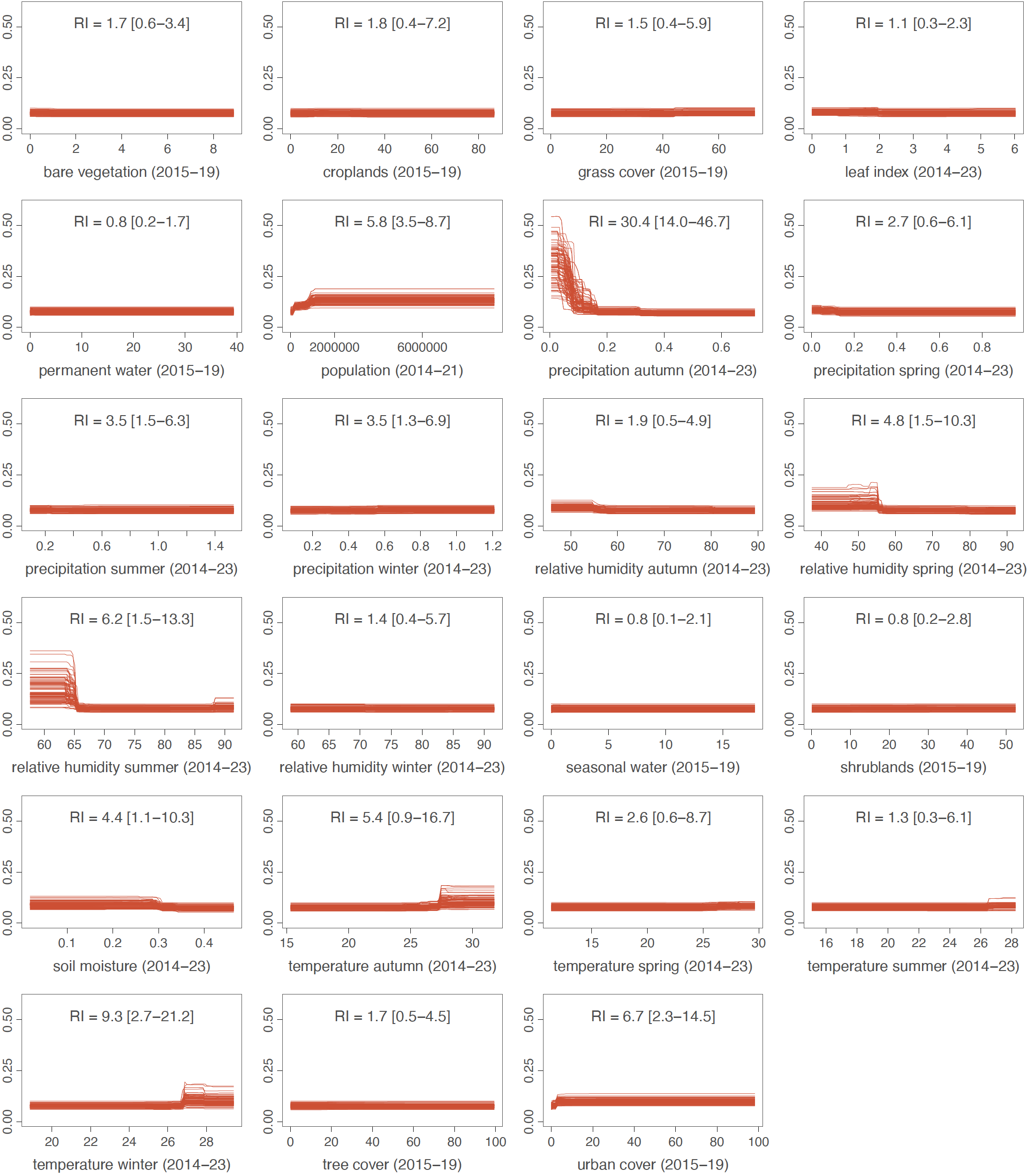
**

**Fig. S2.** **Partial dependency plots of the ecological niche models.** For each environmental factor, we generated partial dependency curves for all 100 replicates boosted regression tree (BRT) models. These curves illustrate how each environmental variable (shown on the x-axis) impacts the response variable, which is the ecological suitability for WNV (shown on the y-axis). The curves were created by analyzing the variation in ecological suitability associated with each specific environmental variable while holding all other variables constant at their median values. The environmental variables examined include bare vegetation, croplands, grass cover, leaf index, human population density (log-transformed), permanent water, precipitation by season, relative humidity by season, temperature by season, seasonal water, shrublands, soil moisture, tree cover, and urban cover.

**Supplementary Table**

**Table S1.** The testing positivity rate of West Nile virus by year between January 1, 2014, and December 31, 2024.

| **Year** | **Number of positive cases** | **Number of tested cases** | **Testing positivity rate** |
| --- | --- | --- | --- |
| 2014 | 6 | 318 | 1.8868% |
| 2015 | 1 | 409 | 0.2445% |
| 2016 | 0 | 0 | 0% |
| 2017 | 3 | 9,492 | 0.0316% |
| 2018 | 12 | 8,515 | 0.1409% |
| 2019 | 19 | 36,890 | 0.0515% |
| 2020 | 13 | 13,003 | 0.0999% |
| 2021 | 22 | 9,404 | 0.2339% |
| 2022 | 2 | 15,086 | 0.0133% |
| 2023 | 26 | 26,057 | 0.0998% |
| 2024 | 6 | 73,175 | 0.0082% |
| **Overall** | **110** | **194,445** | **0.0566%** |

**Table S2.** Primers and probes used for the detection of arboviruses in this study.

| **Virus** | **Sequences (5’→3’)** | **Primers and probes** | **Target** | **Genome position** | **Ref.** |
| --- | --- | --- | --- | --- | --- |
| WNV | CAGACCACGCTACGGCG | Forward | E | 10668-10684 | ^2^ |
|  | CTAGGGCCGCGTGGG | Reverse | E | 10770-10756 |  |
|  | TCTGCGGAGAGTGCAGTCTGCGAT | Probe | E | 10691-10714 |  |
| DENV1 | GACACCACACCCTTTGGACAA | Forward | NS5 | 8586-8606 | ^5^ |
|  | CACYTGGGCTGTCACCTCCAT | Reverse | NS5 | 8692-8673 |  |
|  | AGAGGGTGTTTAAAGAGAAAGTTGACACGCG | Probe |  | 8608-8638 |  |
| DENV2 | CAGGTTATGGCACTGTCACGAT | Forward | M | 1605 | ^4^ |
|  | CCATCTGCAGCAACACCATCTC | Reverse | M | 1583 |  |
|  | CTCCGAGAACAGGCCTCGACTTCAA | Probe |  | 1008 |  |
| DENV3 | GGGAAAACCGTCTATCAATA | Forward | C | 118-221 | ^5^ |
|  | CGCCATAACCAATTTCATTGG | Reverse | C | 241-221 |  |
|  | CACAGTTGGCGAAGAGATCTCAAGAGGA | Probe |  | 174-202 |  |
| DENV4 | TGAAGAGATTCTCAACCGGAC | Forward | C | 187-207 | ^5^ |
|  | AATCCCTGCTGTTGGTGGC | Reverse | C | 293-275 |  |
|  | TCATCACGTTTTTGCGAGTCCTTTCCA | Probe |  | 247-273 |  |
| CHIKV | AAAGGGCAAACTCAGCTTCAC | Forward | NSP1 | 874-894 | ^3^ |
|  | GCCTGGGCTCATCGTTATTC | Reverse | NSP1 | 961-942 |  |
|  | CGCTGTGATACAGTGGTTTCGTGTG | Probe |  | 899-923 |  |

Legend: WNV, West Nile virus. DENV, dengue virus. CHIKV, chikungunya virus. E, envelope. NS5, non-structural protein 5. M, matrix protein. C, capsid protein. NSP1, non-structural protein 1.

**Table S3.** The number of samples tested and the positive rate for WNV in Ceará State, Brazil.

| **Year Month** | **Samples tested** | **Positive sample** | **Positivity rate (%)** |
| --- | --- | --- | --- |
| 2019-01 | 4 | 1 | 25·0 |
| 2019-02 | 3 | 0 | 0 |
| 2019-03 | 1 | 0 | 0 |
| 2019-04 | 3 | 0 | 0 |
| 2019-05 | 5 | 0 | 0 |
| 2019-06 | 2 | 0 | 0 |
| 2019-07 | 2 | 0 | 0 |
| 2019-08 | 2 | 0 | 0 |
| 2019-09 | 10 | 2 | 20 |
| 2019-10 | 11 | 0 | 0 |
| 2019-11 | 13 | 3 | 23·1 |
| 2019-12 | 9 | 2 | 22·2 |
| 2020-01 | 11 | 2 | 18·2 |
| 2020-02 | 19 | 1 | 5·3 |
| 2020-03 | 11 | 3 | 27·3 |
| 2020-04 | 5 | 1 | 20·0 |
| 2020-05 | 2 | 0 | 0 |
| 2020-06 | 24 | 1 | 4·2 |
| 2020-07 | 13 | 1 | 7·7 |
| 2020-08 | 6 | 0 | 0 |
| 2020-09 | 13 | 0 | 0 |
| 2020-10 | 14 | 0 | 0 |
| 2020-11 | 7 | 1 | 14·3 |
| 2020-12 | 6 | 0 | 0 |
| 2021-01 | 3 | 1 | 33·3 |
| 2021-02 | 4 | 0 | 0 |
| 2021-03 | 14 | 1 | 7·1 |
| 2021-04 | 9 | 0 | 0 |
| 2021-05 | 8 | 0 | 0 |
| 2021-06 | 12 | 2 | 16·7 |
| 2021-07 | 18 | 0 | 0 |
| 2021-08 | 18 | 1 | 5·6 |
| 2021-09 | 4 | 1 | 25·0 |
| 2021-10 | 4 | 0 | 0 |
| 2021-11 | 1 | 0 | 0 |
| 2021-12 | 0 | 0 | 0 |
| 2022-01 | 5 | 0 | 0 |
| 2022-02 | 8 | 1 | 12·5 |
| 2022-03 | 7 | 0 | 0 |
| 2022-04 | 10 | 2 | 20·0 |
| 2022-05 | 11 | 0 | 0 |
| 2022-06 | 14 | 1 | 7·1 |
| 2022-07 | 18 | 3 | 16·7 |
| 2022-08 | 11 | 1 | 9·1 |
| 2022-09 | 15 | 1 | 6·7 |
| 2022-10 | 14 | 3 | 21·4 |
| 2022-11 | 5 | 0 | 0 |
| 2022-12 | 12 | 2 | 16·7 |
| 2023-01 | 7 | 1 | 14·3 |
| 2023-02 | 11 | 4 | 36·4 |
| 2023-03 | 17 | 4 | 23·5 |
| 2023-04 | 16 | 6 | 37·5 |
| 2023-05 | 9 | 0 | 0 |
| 2023-06 | 9 | 1 | 11·1 |
| 2023-07 | 12 | 1 | 8·3 |
| 2023-08 | 11 | 1 | 9·1 |
| 2023-09 | 12 | 1 | 8·3 |
| 2023-10 | 3 | 2 | 66·7 |
| 2023-11 | 23 | 6 | 26·1 |
| 2023-12 | 3 | 2 | 66·7 |
| 2024-01 | 1 | 1 | 100 |

**Table S4.** Genome sequences used in the phylogenetic analysis.

| **Accession** | **Country** | **Host** | **Year** |
| --- | --- | --- | --- |
| PQ438738 | Brazil | *Equus caballus* | 2019 |
| OP422646 | Brazil | Culex | 2017 |
| ON782108 | Brazil | *Equus caballus* | 2018 |
| ON782109 | Brazil | *Equus caballus* | 2019 |
| MT905060 | Brazil | *Equus caballus* | 2019 |
| MT863559 | France | *Equus caballus* | 2015 |
| MH643887 | Brazil | *Equus caballus* | 2018 |
| MH166886 | USA | Culicidae | 2001 |
| MH166897 | USA | Culicidae | 2010 |
| MH166899 | USA | Culicidae | 2002 |
| MH170242 | USA | Culicidae | 2007 |
| MH170263 | USA | *Cyanocitta cristata* | 2001 |
| MH170273 | USA | *Corvus brachyrhynchos* | 2001 |
| MH170276 | USA | *Corvus brachyrhynchos* | 2001 |
| KY703854 | Senegal | *Culex poicilipes* | 1990 |
| KU978766 | Colombia | *Phoenicopterus ruber* | 2008 |
| KU978769 | Mexico | Corvus | 2003 |
| KX547252 | USA | Culiseta | 2002 |
| KX547253 | USA | *Corvus brachyrhynchos* | 2004 |
| KX547301 | USA | Culiseta | 2002 |
| KX547322 | USA | Culiseta | 2003 |
| KX547414 | USA | Culiseta | 2002 |
| KX547428 | USA | Culiseta | 2002 |
| JX442279 | China | *Culex pipiens* | 2011 |
| KJ501233 | USA | *Cyanocitta cristata* | 2001 |
| KJ501234 | USA | *Cyanocitta cristata* | 2001 |
| KJ501235 | USA | *Cyanocitta cristata* | 2001 |
| KJ501264 | USA | *Cyanocitta cristata* | 2001 |
| KJ501268 | USA | Falco | 2001 |
| KJ501275 | USA | *Corvus brachyrhynchos* | 2001 |
| KJ501282 | USA | *Bubo virginianus* | 2001 |
| KJ501317 | USA | *Corvus brachyrhynchos* | 2001 |
| KJ501320 | USA | *Corvus brachyrhynchos* | 2003 |
| KJ501332 | USA | *Corvus brachyrhynchos* | 2001 |
| KJ501362 | USA | *Corvus brachyrhynchos* | 2001 |
| KJ501363 | USA | *Corvus brachyrhynchos* | 2001 |
| KJ501379 | USA | *Cyanocitta cristata* | 2001 |
| KJ501393 | USA | *Corvus brachyrhynchos* | 2001 |
| KJ501394 | USA | *Corvus brachyrhynchos* | 2001 |
| KJ501395 | USA | *Corvus brachyrhynchos* | 2001 |
| KJ501443 | USA | *Corvus brachyrhynchos* | 2001 |
| KJ501455 | USA | *Cyanocitta cristata* | 2001 |
| KJ501468 | USA | Sturnidae | 2001 |
| KJ501473 | USA | *Corvus brachyrhynchos* | 2001 |
| KJ501489 | USA | *Corvus brachyrhynchos* | 2002 |
| KJ501492 | USA | *Pelecanus erythrorhynchos* | 2005 |
| KJ501512 | USA | *Corvus brachyrhynchos* | 2001 |
| KJ501516 | USA | *Corvus brachyrhynchos* | 2001 |
| KF234080 | Italy | *Homo sapiens* | 2009 |
| JX041629 | Azerbaijan | Aves | 1967 |
| JX041630 | Azerbaijan | Aves | 1967 |
| JN051152 | Mexico | *Corvus corax* | 2003 |
| JN051153 | Mexico | *Corvus corax* | 2003 |
| JF719069 | Spain | *Equus caballus* | 2010 |
| HM488218 | USA | *Culex pipiens* | 2003 |
| HM488236 | USA | *Culiseta melanura* | 2003 |
| HM152773 | Israel | *Homo sapiens* | 2000 |
| GU011992.2 | Italy | *Homo sapiens* | 2009 |
| GQ379156 | USA | Corvus | 2001 |
| GQ379161 | Argentina | *Equus caballus* | 2006 |
| DQ983578 | USA | *Culex nigripalpus* | 2003 |
| DQ431697 | USA | *Homo sapiens* | 2003 |
| DQ080062 | USA | Culicidae | 2002 |
| DQ080071 | Mexico | *Equus caballus* | 2002 |
| DQ080072 | USA | Dumetella | 2001 |
| DQ164202 | USA | *Homo sapiens* | 2002 |
| AY660002 | Mexico | Corvus | 2003 |

**References**

1. Brasil-Ministério-da-Saúde-Secretaria-de-Vigilância-em-Saúde. Manual de Vigilância Sentinela  de Doenças Neuroinvasivas por Arbovírus. Brasília, DF: Ministério da Saúde, 2017.; 2017. p. 44.

2. Lanciotti RS, Kerst AJ, Nasci RS, et al. Rapid detection of west nile virus from human clinical specimens, field-collected mosquitoes, and avian samples by a TaqMan reverse transcriptase-PCR assay. *J Clin Microbiol* 2000; **38**(11): 4066-71.

3. Lanciotti RS, Kosoy OL, Laven JJ, et al. Chikungunya virus in US travelers returning from India, 2006. *Emerg Infect Dis* 2007; **13**(5): 764-7.

4. Johnson BW, Russell BJ, Lanciotti RS. Serotype-specific detection of dengue viruses in a fourplex real-time reverse transcriptase PCR assay. *J Clin Microbiol* 2005; **43**(10): 4977-83.

5. Callahan JD, Wu SJ, Dion-Schultz A, et al. Development and evaluation of serotype- and group-specific fluorogenic reverse transcriptase PCR (TaqMan) assays for dengue virus. *J Clin Microbiol* 2001; **39**(11): 4119-24.

6. Lanciotti RS, Kosoy OL, Laven JJ, et al. Genetic and serologic properties of Zika virus associated with an epidemic, Yap State, Micronesia, 2007. *Emerg Infect Dis* 2008; **14**(8): 1232-9.

7. Quick J, Grubaugh ND, Pullan ST, et al. Multiplex PCR method for MinION and Illumina sequencing of Zika and other virus genomes directly from clinical samples. *Nat Protoc* 2017; **12**(6): 1261-76.

8. Li H. Minimap2: pairwise alignment for nucleotide sequences. *Bioinformatics* 2018; **34**(18): 3094-100.

9. Li H, Handsaker B, Wysoker A, et al. The Sequence Alignment/Map format and SAMtools. *Bioinformatics* 2009; **25**(16): 2078-9.

10. Katoh K, Standley DM. MAFFT multiple sequence alignment software version 7: improvements in performance and usability. *Mol Biol Evol* 2013; **30**(4): 772-80.

11. Martin DP, Varsani A, Roumagnac P, et al. RDP5: a computer program for analyzing recombination in, and removing signals of recombination from, nucleotide sequence datasets. *Virus Evol* 2021; **7**(1): veaa087.

12. Minh BQ, Schmidt HA, Chernomor O, et al. IQ-TREE 2: New Models and Efficient Methods for Phylogenetic Inference in the Genomic Era. *Mol Biol Evol* 2020; **37**(5): 1530-4.

13. Kalyaanamoorthy S, Minh BQ, Wong TKF, von Haeseler A, Jermiin LS. ModelFinder: fast model selection for accurate phylogenetic estimates. *Nat Methods* 2017; **14**(6): 587-9.

14. Hoang DT, Chernomor O, von Haeseler A, Minh BQ, Vinh LS. UFBoot2: Improving the Ultrafast Bootstrap Approximation. *Mol Biol Evol* 2018; **35**(2): 518-22.

15. Rambaut A, Lam TT, Max Carvalho L, Pybus OG. Exploring the temporal structure of heterochronous sequences using TempEst (formerly Path-O-Gen). *Virus Evol* 2016; **2**(1): vew007.

16. Suchard MA, Lemey P, Baele G, Ayres DL, Drummond AJ, Rambaut A. Bayesian phylogenetic and phylodynamic data integration using BEAST 1.10. *Virus Evol* 2018; **4**(1): vey016.

17. Gill MS, Lemey P, Faria NR, Rambaut A, Shapiro B, Suchard MA. Improving Bayesian population dynamics inference: a coalescent-based model for multiple loci. *Mol Biol Evol* 2013; **30**(3): 713-24.

18. Baele G, Gill MS, Lemey P, Suchard MA. Hamiltonian Monte Carlo sampling to estimate past population dynamics using the skygrid coalescent model in a Bayesian phylogenetics framework. *Wellcome Open Res* 2020; **5**: 53.

19. Ayres DL, Darling A, Zwickl DJ, et al. BEAGLE: an application programming interface and high-performance computing library for statistical phylogenetics. *Syst Biol* 2012; **61**(1): 170-3.

20. Serres K. ED, Despréaux G., Vicenti-González MF., Van-Bortel W.. Arsevska E., Dellicour, S. Integrating indicator-based and event-based surveillance data to enhance vector-borne

disease risk mapping in Europe. Eurosurveillance; 2024.

21. Giesen C, Herrador Z, Fernandez-Martinez B, et al. A systematic review of environmental factors related to WNV circulation in European and Mediterranean countries. *One Health* 2023; **16**: 100478.
