# Supplementary material for: Active West Nile virus transmission in Brazil: an epidemiological study
Source: Lancet Reg Health Am. 2025 Sep 8;51:101229. doi: 10.1016/j.lana.2025.101229 (PMC12448031; doi:10.1016/j.lana.2025.101229)
Supplement: Abstract_Portuguese_final [file mmc2.docx]

Editor’s note: This translation in Portuguese was submitted by the authors and we reproduce it as supplied. It has not been peer reviewed. Our editorial processes have only been applied to the original abstract in English, which should serve as reference for this manuscript.

**Transmissão ativa do vírus da Febre do Nilo Ocidental no Brasil: um estudo epidemiológico**

**Resumo**

**Contexto:** O vírus da Febre do Nilo Ocidental (VNO) é um flavivírus transmitido por mosquitos que pode causar doenças neurológicas e fatais em animais e humanos. Desde sua introdução nos Estados Unidos da Américas em 1999, o VNO tornou-se o principal arbovírus na América do Norte. Em contraste, nenhum grade surto de VNO foi descrito na América do Sul. Nosso estudo investigou a circulação ativa do VNO no Brasil.

**Métodos:** Examinamos dados epidemiológicos, moleculares, genômicos e sorológicos do VNO no Brasil, de janeiro de 2014 a dezembro de 2024. Também realizamos testes para VNO em 561 pacientes com doença febril, doença neuroinvasiva ou óbito entre janeiro de 2019 e janeiro de 2024 no Ceará, Brasil. Em seguida, realizamos séries temporais, mapeamento, modelagem de nicho ecológico, distribuição por idade e sexo, análises filogenéticas e testes de hipóteses estatísticas.

**Resultados:** Entre janeiro de 2014 e dezembro de 2024, 110 casos de febre do Nilo Ocidental foram notificados em 13 dos 27 estados brasileiros. Além disso, nosso estudo retrospectivo no Ceará revelou que 12,1% (68 de 561 pacientes) eram casos de VNO, com pico em 2023, quando ocorreram 42,6% (29 de 68) dos casos. Entre os casos de VNO, 7 (10,3%) apresentaram RNA de VNO detectado no soro, líquido cefalorraquidiano ou ambos, enquanto 62 (89,7%) eram IgM positivos, com 29 apresentando complicações neurológicas, 35 com doença febril e quatro mortes. Os casos de VNO foram notificados em todos os meses, com os maiores números entre maio e agosto. A maioria dos casos foi em mulheres (razão mulher-homem, 1,1:1), e a mediana de idade dos pacientes foi de 40 anos (intervalo interquartil, 20-57). Nossa análise filogenética mostrou que a linhagem 1a do VNO circulou Ceará e causou um caso fatal em um cavalo. Nossos modelos de nicho ecológico identificaram diversas áreas, principalmente na região Nordeste, associadas a um risco potencialmente maior de exposição humana à circulação local do VNO.

**Interpretação:** Essas descobertas descreveram de forma abrangente a circulação consistente do VNO no Brasil e podem contribuir para informar as políticas de saúde pública, com foco nas estratégias para determinar o impacto do VNO na América do Sul.

**Financiamento:** *Burroughs Wellcome Fund*, *Wellcome Trust*, *US National Institutes of Health*, Fundação de Amparo à Pesquisa do Estado de São Paulo, e Conselho Nacional de Desenvolvimento Científico e Tecnológico.
